# Reconstruction of ancestral gene orders using intermediate genomes

**DOI:** 10.1186/1471-2105-16-S14-S3

**Published:** 2015-10-02

**Authors:** Pedro Feijão

**Affiliations:** 1 Technische Fakultät and CeBiTec, Universität Bielefeld, Universitätsstr. 25, 33615 Bielefeld, Germany

**Keywords:** Ancestral Reconstruction, Small Parsimony Problem, Genome Rearrangement, Double-Cut-and-Join

## Abstract

**Background:**

The problem of reconstructing ancestral genomes in a given phylogenetic tree arises in many different comparative genomics fields. Here, we focus on reconstructing the gene order of ancestral genomes, a problem that has been largely studied in the past 20 years, especially with the increasing availability of whole genome DNA sequences. There are two main approaches to this problem: event-based methods, that try to find the ancestral genomes that minimize the number of rearrangement events in the tree; and homology-based, that look for conserved structures, such as adjacent genes in the extant genomes, to build the ancestral genomes.

**Results:**

We propose algorithms that use the concept of *intermediate genomes*, arising in optimal pairwise rearrangement scenarios. We show that intermediate genomes have combinatorial properties that make them easy to reconstruct, and develop fast algorithms with better reconstructed ancestral genomes than current event-based methods. The proposed framework is also designed to accept extra information, such as results from homology-based approaches, giving rise to combined algorithms with better results than the original methods.

## Background

Reconstructing ancestral genomes based on gene order data is an important problem that has been largely studied in the past years, especially with the increasing availability of whole genome DNA sequences. This problem, also called the *small parsimony problem*, receives as input a phylogenetic tree with extant genomes at its leaves, and asks for the reconstructed gene orders at the internal nodes of the tree, corresponding to ancestral genomes.

There are two main approaches for solving this problem. The first is a parsimonious approach, that will be called *event*- or *distance-based*, that assumes a model of rearrangement and reconstructs the ancestral genomes that minimize the total number of rearrangement events on the tree, or said differently, the total tree length, as measured by the rearrangement distance.

The first proposed method using the distance-based approach, BPAnalysis [1], used the breakpoint distance. With the development of better rearrangement models, such as signed reversals and the Double-Cut-and-Join (DCJ) distance [2,3], new algorithms have been proposed. Notable examples of recent distance-based methods include MGRA [4], PATHGROUPS [5], and GASTS [6], based on minimizing the DCJ distance, and SCJ Small Phylogeny [7], based on the Single-Cut-or-Join (SCJ) model [8]. Since rearrangement distance problems are usually NP-hard for three or more genomes, these methods tend to be time consuming, especially when exact solutions are implemented.

On the other hand, homology methods do not use rearrangement models, but instead look for conserved structures between the input genomes, such as conserved adjacencies or gene clusters, and use this information to assemble Contiguous Ancestral Regions (CARs), as pioneered by Ma *et al*. in InferCARs [9], an approach explored by many recent algorithms, such as GapAdj [10], ANGES [11], PMAG [12], MLGO [13] and ProCARs [14].

In this paper, a new approach is proposed, inspired by combinatorial ideas from rearrangement models, though not directly motivated as a distance-based method. Specifically, we propose the use of *intermediate genomes*, arising from optimal rearrangement scenarios between two genomes. The motivation is the fact that, if we consider an internal node in a phylogenetic tree, corresponding to a speciation event, then by the parsimony principle, this internal node should be an intermediate genome in a optimal rearrangement scenario between the two genomes at its children nodes. Yet, most current parsimonious approaches focus only on minimizing events on the tree, without extra restrictions.

This paper is organized as follows. First, we characterize all possible intermediate genomes in a parsimonious path between two genomes, and show that using the restriction that internal nodes must be intermediate genomes improves the ancestral reconstruction results, even though the trees do not minimize the number of events. The proposed framework can easily accept additional information, such as results from homology-based methods, giving rise to combined algorithms that have better results than the original methods.

## Preliminaries

**Figure 1:**
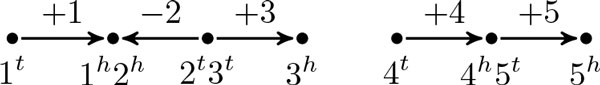
**Genome *G *= {(○ 1 −2 3 ○), (○ 4 5 ○)} with adjacency set *A *= {○1*^t^*, 1*^h ^*2*^h^*, 2*^t ^*3*^t^*, 3*^h ^*○, ○4*^t^*, 4*^h ^*5*^t^*, 5*^h^*○}**.

We represent multichromosomal genomes using a similar notation as in previous works [3,15]. A *gene *is an oriented fragment of DNA represented by a signed integer, where the sign denotes the orientation. A *chromosome *is represented by a sequence of genes, flanked in the extremities by *telomeres *(○) if the chromosome is linear; otherwise, it is circular. *Genomes *are sets of chromosomes, where each gene occurs exactly once. A gene *g *has two *extremities*, the *tail *(*g^t ^*) and the *head *(*g^h ^*). An *adjacency *in a genome is an unordered pair of either consecutive gene extremities in a chromosome, or a gene extremity with a telomere (called *telomeric adjacency *). A genome *G_A _*is represented either by the set of chromosomes, or also by the set of adjacencies *A*. For instance, the genome *G_A _*of Figure 1 has two linear chromosomes and five genes, with *G*_*A *_= {(○ 1 −2 3 ○), (○ 4 5 ○)} and *A *= {○1*^t^*, 1*^h ^*2*^h^*, 2*^t ^*3*^t^*, 3*^t^*○, ○4*^t^*, 4*^h ^*5*^t^*, 5*^h ^*○}.

**Figure 2:**
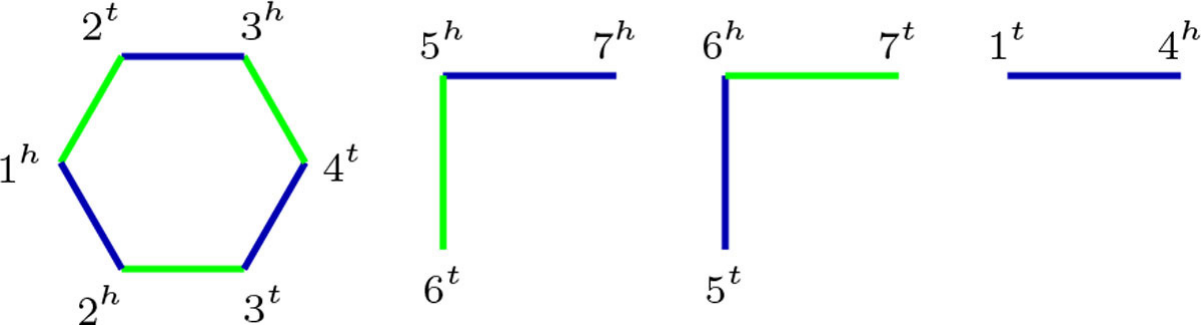
**Breakpoint graph BP(*A, B*) of *A *= {○1*^t^*, 1*^h ^*2*^t^*, 2*^h ^*3*^t^*, 3*^h ^*4*^t^*, 4*^h ^*○, ○5*^t^*, 5*^h^*6*^t^*, 6*^h ^*7*^t^*, 7*^h ^*○} and *B *= {1*^h ^*2*^h^*, 2*^t ^*3*^h^*, 3*^t ^*4*^t^*, 4*^h ^*1*^t^*, ○6*^t^*, 6*^h ^*5*^t^*, 5*^h ^*7*^h^*, 7*^t^*○}**. *A*-edges are drawn in green, and *B*-edges in blue.

Given two genomes *A *and *B *with the same set of genes, the *breakpoint graph *[16] of *A *and *B*, denoted by BP(*A, B*), is a graph *G *where the vertex set *V *(*G*) is the set of gene extremities, and the edge set *E*(*G*) is the set of non-telomeric adjacencies of *A *and *B*, called *A*-edges and *B*-edges, respectively. Adjacencies from *A *an *B *are usually drawn with different colors, as seen in Figure 2. A connected component in BP(*A, B*) is always a color alternating component and will be called *AB-component*. The *size *of an AB-component is the number of vertices it has. An AB-component is either a cycle, an even path or an odd path, where the parity of a path is based on the number of *vertices *of the path (an isolated vertex is considered an odd path). Note that the parity is usually defined on edges, not vertices, but here the vertex parity is more natural.

The *Double-Cut-and-Join *(DCJ) [2,3] operation rearranges genes in a genome by cutting two adjacencies and rejoining the extremities in a different way. For instance, adjacencies *pq *and *rs *become *pr *and *qs*, or *p*○ and *qr *become *pr *and *q*○. It is also possible to cut an adjacency *pq *into *p*○ and *q*○, and the reverse operation.

The *DCJ distance d*(*A, B*) between genomes *A *and *B *is defined as the minimum number of DCJ operations needed to transform *A *into *B*. A *sorting scenario * S between two genomes *A *and *B *is an ordered list of genomes  S = (*M*_0 _, *M*_1 _,..., *M_k _*) where *k *= *d*(*A, B*), *A *= *M*_0 _, *M_k _*= *B *and *M*_*i *_can be obtained from *M*_*i*-1 _by applying a DCJ operation, for *i *= 1,..., *k*. Any genome *M_i _*∈  S is called an *intermediate genome *of *A *and *B*.

The DCJ distance *d*(*A, B*) can be determined using the Breakpoint Graph BP(*A, B*) [3]:

(1) $$d\left(A,B\right)=n-c-\frac{o}{2}$$

where *n *is the number of genes, *c *and *o *are the number of cycles and odd paths in BP(*A, B*), respectively. The problem of transforming *A *into *B *is then the problem of finding DCJ operations that increase the number of cycles and odd paths in BP(*A, B*).

Another way of determining the DCJ distance from BP(*A, B*) is considering the *sorting cost *of each AB-component independently [8,15]. An AB-component with *n *vertices has cost *n*/2 − 1 if it is a cycle, (*n *− 1)/2 if it is an odd path and *n*/2 if it is an even path. The sorting cost of a component *C *will be denoted as *d*(*C*). In the example of Figure 2, using equation (1) gives *d*(*A, B*) = 7 − 1 − 2/2 = 5, and summing the contributions of each component from left to right results in *d*(*A, B*) = 2 + 1 + 1 + 1 = 5.

**Figure 3:**
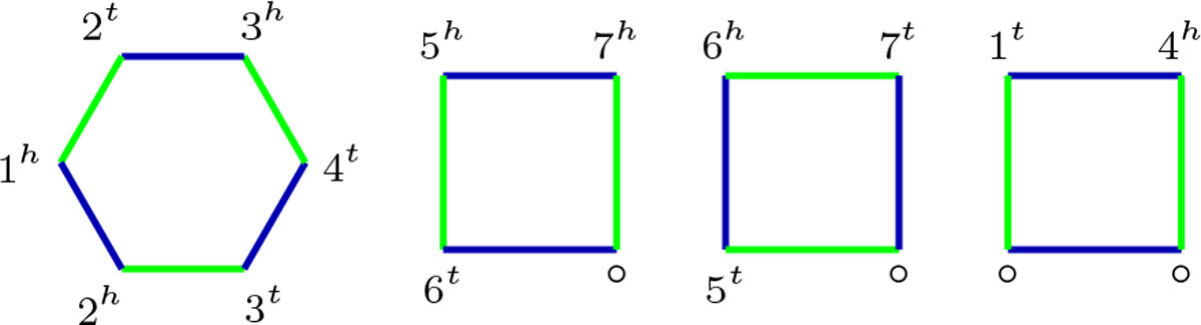
**Circular BP graph BP(*A, B*) of the genomes in **Figure 2**, with telomeric adjacencies**. *A*-edges are drawn in green, and *B*-edges in blue.

It is sometimes useful to include telomeric adjacencies in BP(*A, B*). With similar ideas as used by Braga and Stoye [15], telomere nodes are added in the Breakpoint Graph as follows: for each odd path, add one telomere and connect both telomeric adjacencies of *A *and *B *of the odd path to this new telomere node. For each even path, since both telomeric adjacencies are from the same genome, add two telomere nodes, connecting each adjacency with a different telomere, also adding and edge between both telomeres, representing what is usually called a *null chromosome *on the other genome. This extended version can be called *Circular Breakpoint Graph*, because it has only cycles, as we can see in Figure 3. The Circular Breakpoint Graph has the advantage that every AB-component is a cycle (called an *AB-cycle*), and any DCJ operation is equivalent to removing two edges of the same color (same genome) and replacing with two new edges that reconnect the four vertices in a different way. Another property of the Circular BP(*A, B*) is that the sorting cost of each component preserved. An odd path with *n *vertices has cost (*n *− 1)/2 in the original BP(*A, B*). With the added telomere, it becomes a cycle with *n*+1 vertices, with cost (*n *+ 1)/2 − 1 = (*n *− 1)/2. Similarly, an even path with *n *vertices has original cost *n*/2, and with the two added telomeres it becomes a cycle with *n *+ 2 vertices, with cost (*n *+ 2)/2 − 1 = *n*/2. Therefore, determining the DCJ distance using the circular BP(*A, B*) components is slightly simpler, as only cycles are present. Equation (1) can also be used for the circular BP(*A, B*), with *n *increased by 1 for each pair of telomeres added, since they are new "genes" in the graph.

During the rest of this paper, the term Breakpoint Graph or BP(*A, B*) always refers to the circular version, with telomeric adjacencies. Also, since edges correspond to adjacencies, both terms will be used inter-changeably when in context.

## Intermediate genomes

Intermediate genomes are genomes arising in sorting scenarios between two genomes. Any genome *M_i _*in a sorting scenario  S = (*A *= *M*_0 _, *M*_1_,..., *M_k _*= *B*), where *k *= *d*(*A, B*), is an intermediate genome of *A *and *B*.

The study of intermediate genomes is motivated by the fact that if genomes *A *and *B *share a common ancestor, by the parsimony principle, it is reasonable to expect that their most recent common ancestor is an intermediate genome of *A *and *B*. Therefore, when reconstructing a phylogenetic tree based on gene order and rearrangement events, one possibility is to enforce that any genome corresponding to an internal node on a binary tree must be an intermediate genome of its two children nodes, in a bottom-up approach. One difficulty is that the number of intermediate genomes is potentially huge. To the best of our knowledge there are no studies about the number of intermediate genomes, but the number of possible DCJ sorting scenarios has been determined [15,17], growing exponentially with the distance between the genomes, so one would expect that a similar behavior occurs with intermediate genomes.

Nevertheless, intermediate genomes have nice combinatorial properties, and approaches exploiting this properties can be both simple and fast, at the same time producing better results than previous methods, as it will be shown in the Results section.

### Properties of intermediate genomes

**Figure 4:**
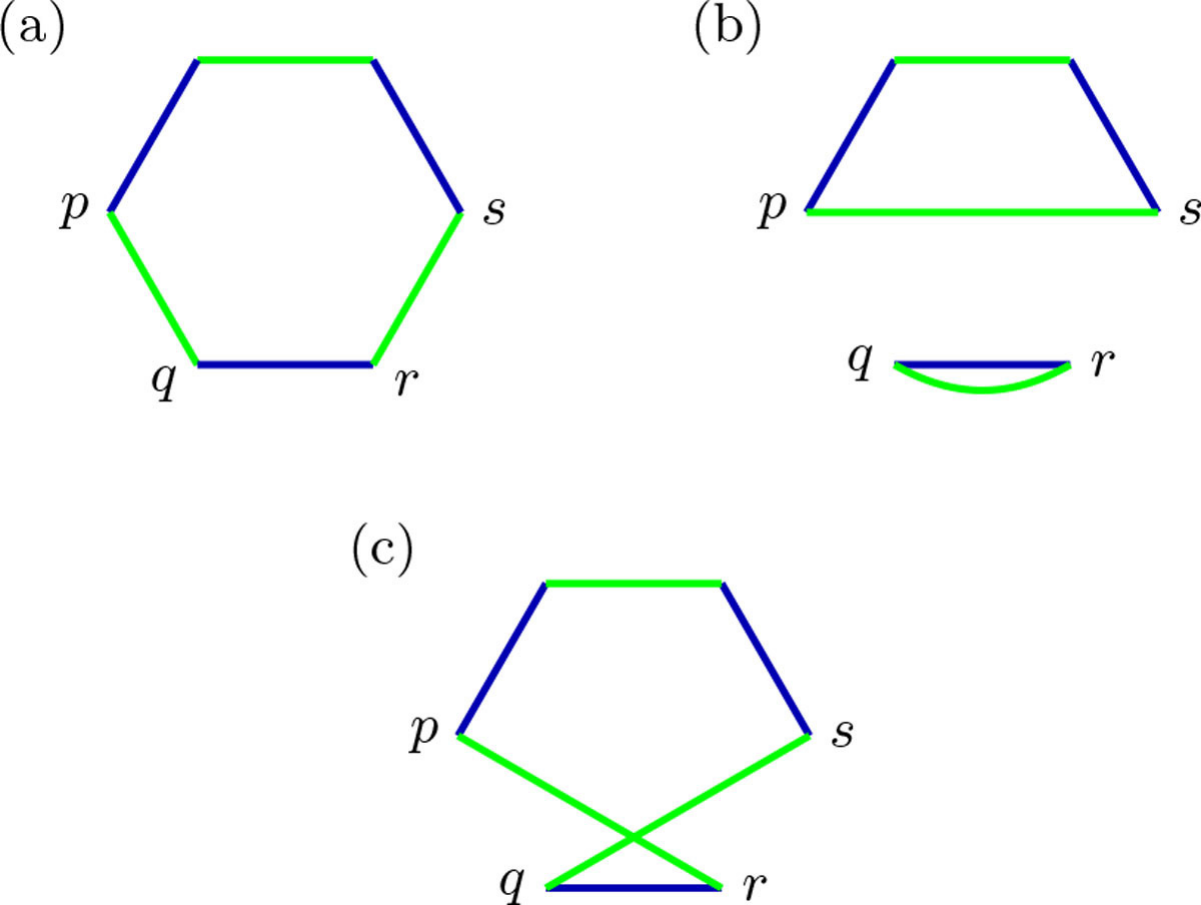
**Possible ways of applying a DCJ operation on adjacencies *pq *and *rs***. (a) The initial BP(*A, B*); (b) Applying DCJ *{pq, rs} → {ps, qr} *on genome *A*; (c) Applying {*pq*, *rs*} → {*pr*, *qs*} on genome *A*; The DCJ in (b) is optimal, increasing the number of cycles by one. The DCJ in (c) is not, and introduces *crossing edges*.

Braga and Stoye showed that it is possible to sort genomes with DCJ operations acting on each component of the Breakpoint Graph independently [15], and from the two possible ways of applying a DCJ in two edges of a component, one is *optimal*, reducing the distance between the genomes, and the other is not, as shown in Figure 4. A scenario that follows this strategy will be called *independent component scenario*. There are scenarios where DCJ operations act on two different components, but these are extremely rare and even non-existing in some cases [15], so they will not be considered here.

**Figure 5:**
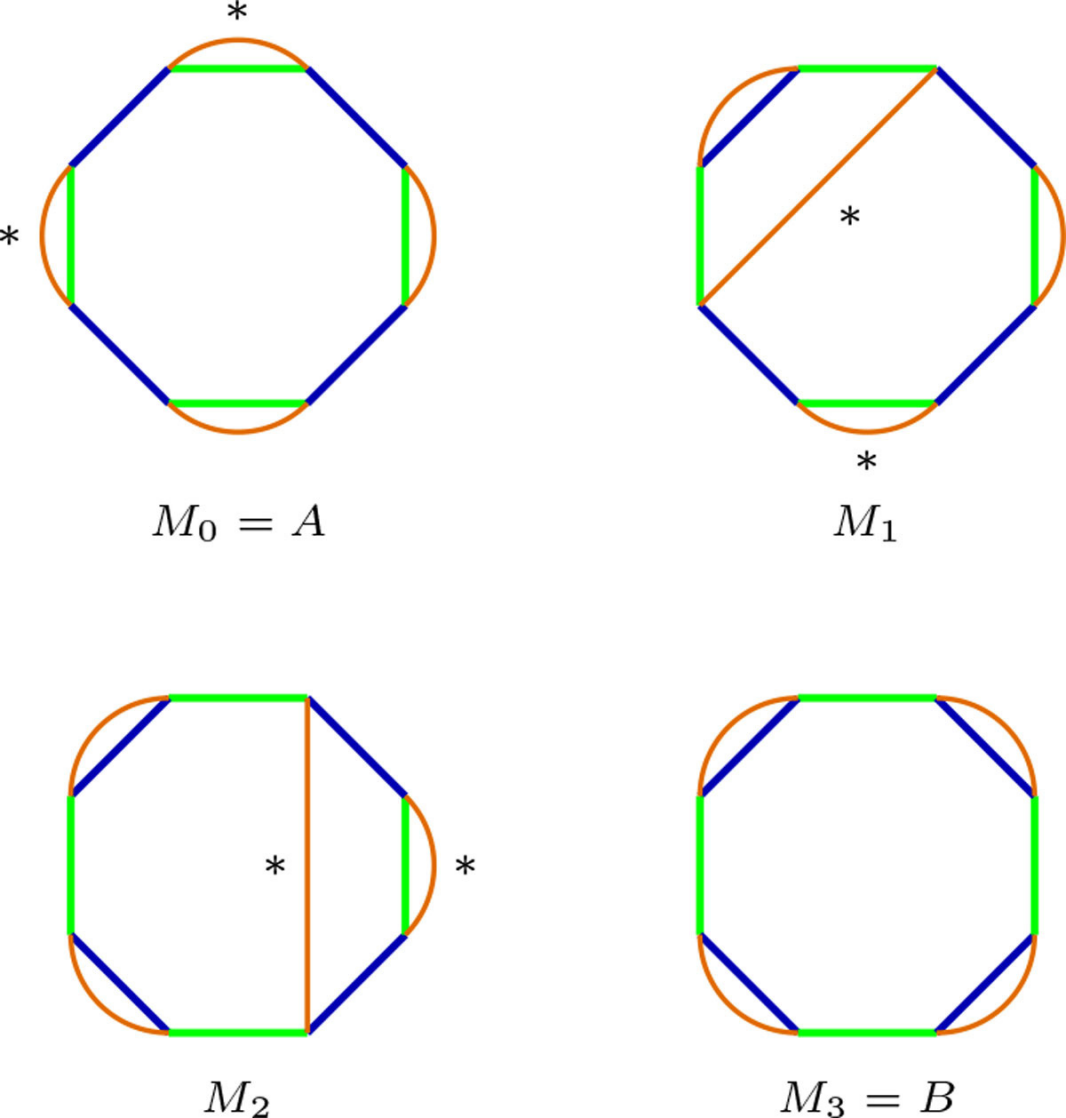
**Scenario ** S**= {*A *= *M*_0 _, *M*_1 _, *M*_2 _, *M*_3 _= *B*} transforming *A *(green edges) into *B *(blue edges) with DCJ operations, shown in BP(*A, B*)**. Intermediate genomes *M_i _*are drawn at each step with orange edges. At each step, a DCJ operation is applied on the edges labeled with ∗. Orange edges never cross in the intermediate genomes.

It is interesting to notice that, since the optimal DCJ breaks a cycle in two, the newly created edges do not intersect. Conversely, in the non-optimal operation, the edges do intersect. This leads to the intuition that the edges of any intermediate genome *M *of *A *and *B*, when drawn on BP(*A, B*), do not cross and do not touch two different components. Such a scenario is shown in Figure 5. This is indeed a general property of intermediate genomes, summarized in the following Theorem.

**Theorem 1 ***A genome M is an intermediate genome of A and B in an independent component scenario if and only if all edges of M are non-crossing chords in the AB-cycles of BP*(*A, B*), *covering all vertices of BP*(*A, B*).

Therefore, the presence of only non-crossing chords is a necessary and sufficient condition for an intermediate genome, in scenarios without component recombination. A similar statement was proven by Ouangraoua and Bergeron [17], where it was shown that optimal DCJ scenarios sorting an AB-cycle are maximal chains in the lattice of *non-crossing partitions *of the cycle.

The proof of Theorem 1 will be derived from a series of small lemmas. The first two prove the necessary condition of the theorem.

**Lemma 1 ***If M is an intermediate genome of A and B, there is no DCJ operation that reduces the distance between M and one of the genomes without increasing the distance to the other*.

*Proof *Assume w.l.g. that there is a DCJ operation transforming *M *into *M' *such that *d*(*M'*, *A*) <*d*(*M*, *A*) and *d*(*M'*, *B*) ≤ *d*(*M*, *B*). Then

$$d\left(M^{\prime },A\right)+d\left(M^{\prime },B\right)<d\left(M,A\right)+d\left(M,B\right)=d\left(A,B\right)$$

breaking the triangle inequality.   □

**Lemma 2 ***If M is an intermediate genome of A and B in an independent component scenario, all edges of M are non-crossing chords in the AB-cycles of BP*(*A, B*), *covering all vertices of BP*(*A, B*).

*Proof *If  S = {*A *= *M*_0 _, *M*_1 _,..., *M_k _*= *B*} is an independent component scenario, there is no *M_i _*in  S that has edges between two AB-components. Also, since each intermediate genome is obtained from the previous one by a DCJ operation, which always removes two edges and creates two new ones with the same vertices, the edges of any *M_i _*cover all vertices of BP(*A, B*).

**Figure 6:**
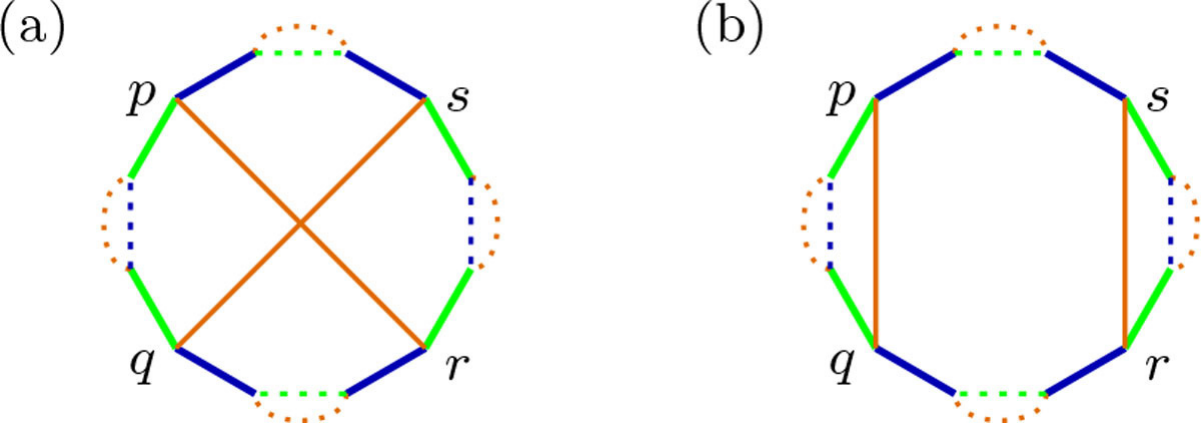
**(a) A genome *M *, in orange, with exactly two crossing edges in BP(*A, B*), with *A *in green and *B *in blue**. (b) A DCJ operation {*pr*, *sq*} → {*pq*, *rs*} on *M *that uncross the edges breaks one AM-cycle in two, keeping the other BM-cycle, meaning that *M *is not an intermediate genome, as shown in Lemma 2.

Now, suppose there is an intermediate genomes in  S with crossing edges. Then, since *M*_0 _= *A *has no crossing edges, there exists a genome *M_i _*, *i *<*k*, where the first crossing occurred. Let *pr *and *sq *be such crossing edges. Since this is the only crossing, between each of the pair of consecutive vertices *p, q, r, s *in the AB-cycle there is an even number of vertices, otherwise the edge adjacent to the odd vertex would have to cross *pr *or *sq*. Then, w.l.g, *p, q, s, r *have to appear consecutively in a AM-cycle, while *p, s, q, r *appear consecutively in a BM-cycle, as shown in Figure 6. Applying the DCJ {*pr*, *qs*} → {*pq*, *sr*} on *M_i _*, the AM-cycle is split in two, while the BM-cycle remains, with different vertex ordering. This means that there is a DCJ operation that decreases *d*(*M_i_*, *A*) while not increasing *d*(*M_i_*, *B*), and by Lemma 1 *M_i _*is not an intermediate genome between *A *and *B*.   □

The next two lemmas prove the sufficient condition of Theorem 1.

**Lemma 3 ***Given an AB-cycle of size *2*n with n noncrossing M-edges covering all vertices, the sum of AM-cycles and BM-cycles is n *+ 1.

**Figure 7:**
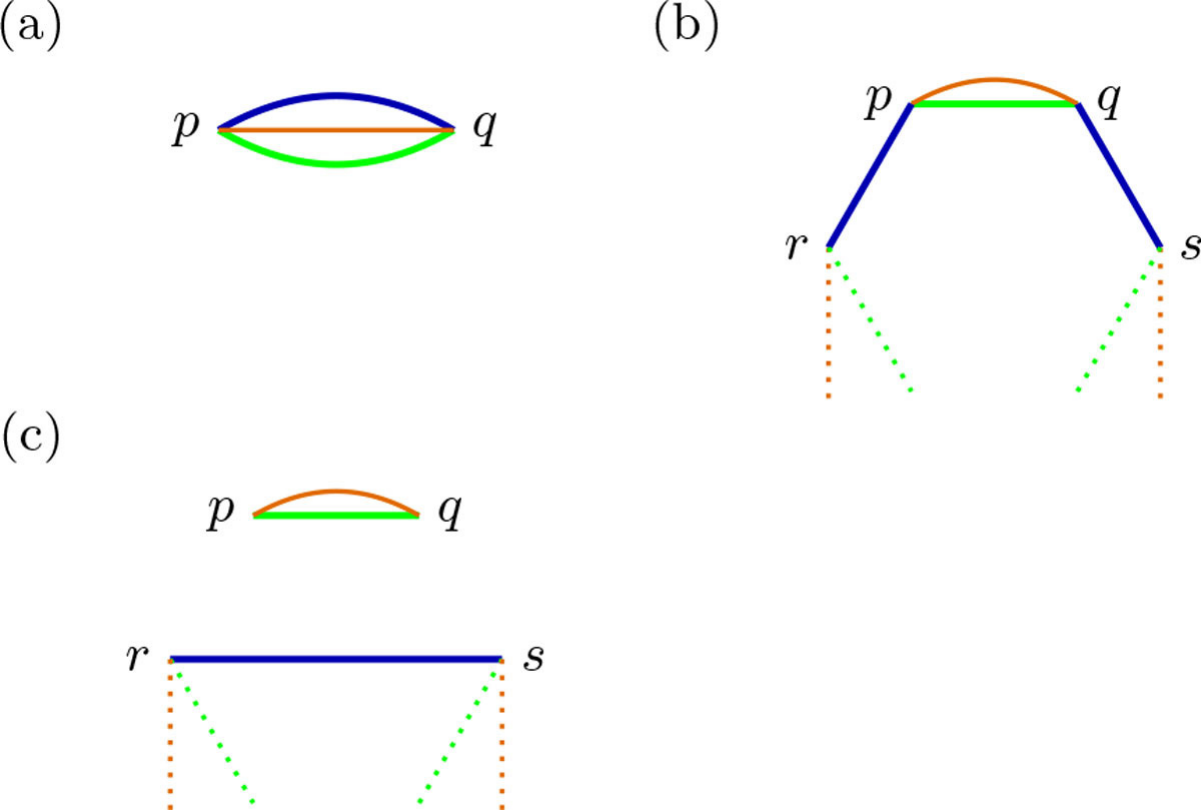
**Number of AM- and BM-cycles in an AB-cycle of size 2*n *with *n *non-crossing M-edges**. *A*-, *B*- and *M *-edges are drawn in green, blue and orange, respectively. (a) An AB-cycle of size 2 has one AM- and one BM-cycle. An AB-cycle of size *n*, shown in (b), has one more AM-cycle than an AB-cycle of size *n *− 2, with the same number of BM-cycles, shown in (c).

*Proof *The proof is by induction on *n*, the number of *M*-edges. Let *C *denote the complete component with the AB-cycle and the M-edges, and let *C_A _*and *C_B _*denote the number of AM-cycles and BM-cycles in a component *C*, respectively. If *n *= 1, then *C_A _*= *C_B _*= 1, and the lemma holds, as seen in Figure 7(a).

For *n *> 1, since the edges of *M *are non-crossing chords covering the entire cycle, *M *has always an edge in common with one of the genomes. W.l.g, let's assume an *A*-edge *e *= *pq *is common between *A *and *M *, and let's call *r *and *s *the vertices adjacent to *p *and *q*, belonging to *B*-edges *rp *and *qs*, as shown in Figure 7(b). Removing the edges *rp *and *qs *and creating a new *B*-edge *rs*, the AM-cycle *pq *is isolated and a new component *C' *with an AB-cycle of size 2(*n *− 1) and *n − *1 M-edges is created, as shown on Figure 7(c). By the inductive hypothesis, since *C' *has *n *− 1 M-edges, $C_{A}^{\prime }+C_{B}^{\prime }=n$. By construction of *C' *, we know that *C *has one extra AM-cycle *pq*, therefore *C_A _*+ *C_B _*= *n *+ 1.

**Lemma 4 ***If the edges of a genome M are noncrossing chords in the AB-cycles of BP*(*A, B*) *covering all vertices of BP*(*A, B*), *M is an intermediate genome of A and B*.

*Proof *From Lemma 3, we know that *C_A _*+ *C_B _*= |*C*|/2 + 1, where *C_A _*and *C_B _*denote the number of *AM *- and *BM *-cycles in a given component *C*, and |*C*| is the number of vertices in *C*. Let *c*_AM _and *c*_BM _denote the total number of *AM *- and *BM *-cycles in all components of BP(*A, B*). Then,

$$\begin{array}{ccc} c_{\text{AM}}+c_{\text{BM}}= & \underset{C\in \text{BP}\left(A,B\right)}{\sum }\left(C_{A}+C_{B}\right) & \\ = & \underset{C\in \text{BP}\left(A,B\right)}{\sum }\left(\frac{\left|C\right|}{2}+1\right)=n+c \end{array}$$

where *n *is the number of genes and *c *the number of AB-cycles in BP(*A, B*). Then,

$$\begin{array}{ccc} d\left(A,M\right)+d\left(B,M\right)= & n-c_{AM}+n-c_{BM}= & \\ = & 2n-\left(n+c\right)=d\left(A,B\right) \end{array}$$

and *M *is and intermediate genome of *A *and *B*.   □

Using Lemmas 4 and 2, Theorem 1 is proved.

### Counting intermediate genomes

Restricted to a given AB-cycle of size 2*n*, the possible choices for the adjacency edges of an intermediate genome *M *correspond to all the ways of finding *n *non-crossing chords in a cycle of size 2*n*. Interestingly, this is given by the ubiquitous *Catalan number *$C_{n}=\frac{1}{n+1}\left(\begin{array}{c} 2n\\ n \end{array}\right)$, as found in sequence A000108 of the On-Line Encyclopedia of Integer Sequences [18]. Therefore, the number of possible intermediate genomes for two genomes *A *and *B*, denoted here as IG(*A, B*), is given by

(2) $$\text{IG}\left(A,B\right)=\underset{K\in \text{BP}\left(A,B\right)}{\prod }C_{|K|/2}$$

where *K *∈ BP(*A, B*) represent all AB-cycles in BP(*A*, *B*), and |*K*| is the number of vertices in a cycle *K*.

## Intermediate genomes for ancestral reconstruction

Given a tree *T *with *n *extant genomes at the leaves, the *ancestral genome reconstruction problem *asks to reconstruct the ancestral genomes corresponding to the internal nodes of the tree.

One proposed solution to this problem, in the context of a rearrangement distance model, is to find ancestral genomes such that the total length of the tree, defined as the sum of all rearrangement distances on the edges, is minimized. This is sometimes called the *small phylogeny problem*, since the tree *T *is given, in contrast to the *large phylogeny problem*, where no tree is given and the aim is to find, from all possible topologies, a tree with minimum length.

In particular, for three genomes *A*, *B *and *C*, this becomes the well studied *genome median problem*, where the aim is to find a genome *M *minimizing *d*(*A*, *M*) + *d*(*B*, *M*) + *d*(*C*, *M*). The median problem is used by many ancestral reconstruction methods as an intermediate step in an iterative approach. Although it is NP-hard for DCJ and many models, it is widely studied, with several exact and heuristic methods already proposed [19,20]. On the other hand, some authors have recently conjectured that it might not be the best option for ancestral inference, since in many instances the solution is one of the input genomes, giving no new information for ancestral reconstruction [21].

Using the concept of intermediate genomes, the following alternative problems are proposed:

**Problem 1 **(Intermediate Genome Small Phylogeny) *Given a binary tree T with n extant genomes at the leaves, find ancestral genomes corresponding to the internal nodes of the tree such that the tree length is minimized, with the restriction that each internal node is an intermediate genome of its children*.

**Problem 2 **(Intermediate Genome Median) *Given two genomes A and B, and an outgroup genome C, find M, an intermediate genome of A and B that minimizes d*(*C*, *M*).

Problem 2 is similar to the median problem, but since *d*(*A*, *M*) + *d*(*B*, *M*) = *d*(*A*, *B*) is constant, it can be left out of the objective function. Therefore, this is the problem of finding an intermediate genome between *A *and *B *that is closest to *C*. A similar problem was studied by Swenson and Moret [22], where they try to find intermediate genomes that are simultaneously in scenarios from *A *to *C *and from *B *to *C*, and are as far as as possible to *C*, meaning that they are close to the input genomes *A *and *B*. In order to do that, it is necessary to find the common intermediate genomes in both scenarios, and this usually leads to an exponential number of scenarios to be considered. Therefore, exact solutions are only found for small genomes, and heuristics have to be used for larger genomes.

The approach proposed here has the advantage that we can detect intermediate genomes without the need to find rearrangement scenarios. The number of intermediate genomes is still exponential, but the use of additional information from the outgroup genome or external input can greatly reduce the search space, as we will see in the next section.

It is likely that the intermediate genome problems proposed here are NP-hard, as their original counterparts, but this remains an open question. Similarly as previous algorithms, one can develop an approach to solve Problem 1 by solving iteratively several instances of Problem 2. But, as a simpler first approach, the solution for the small phylogeny problem presented here is based on a bottom-up procedure, where at each step an internal node is reconstructed as an intermediate genome between its two children, who must be leaves in the tree. In order to do that, instead of minimizing the distance as in Problem 2, we will use the concept of *adjacency guides*.

### Adjacency guides

We propose to solve the following problem:

**Problem 3 **(Guided Intermediate Genome) *Given a set of adjacencies G, called the **adjacency guide**, and two genomes A and B, find an intermediate genome M of A and B using the adjacencies G as a guide*.

This is intentionally a very open definition. What does it mean to use *G *as a guide? One could maximize the number of adjacencies of *G *that are present in *M *, for instance, giving rise to a maximum cardinality independent set problem in a circle graph, that can be solved polynomially. A similar idea was used by Swenson *et al*. [23] in the context of finding the maximum number of non-interfering reversals in the breakpoint graph.

But, more simply, just consider each adjacency *a *∈ *G *sequentially, adding *a *if both vertices belong to the same *AB*-cycle in BP(*A, B*), and it does not cross with previously added adjacencies. Also, *a *has to respect the *parity constraint*, as seen in Lemma 2, where each edge in *M *has to split an AB-cycle in two cycles with even number of vertices.

**Figure 8:**
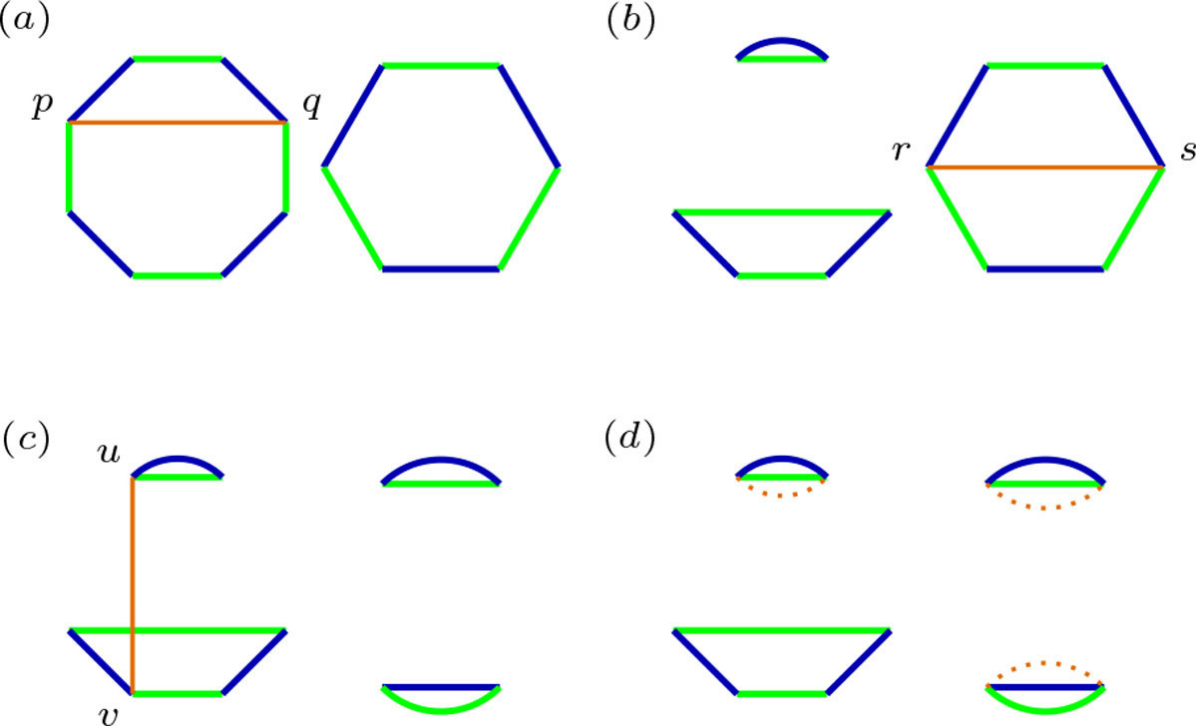
**Solving the Guided Intermediate problem for BP(*A*, *B*) with two AB-cycles, and guide *G *= {*pq*, *rs*, *uv*}**. At each step, an adjacency from *G *is taken, and if accepted its vertices and incident edges are removed, and two new edges are added to complete the resulting two smaller AB-cycles. The problem is then recursively solved on the smaller cycles. (a) Initial BP(*A, B*), where adjacency *pq *is accepted. (b) Second adjacency *rs *is also accepted. (c) The adjacency *uv *covers two components, because it crosses with a previously added adjacency, and it is rejected. (d) In the end of the algorithm, three extra adjacencies (shown as dotted orange edges) are added for each AB-cycle of size 2, since this is the only choice for a non-crossing edge belonging to an intermediate genome *M*. The final output is then the adjacency set {*pq*, *rs*} plus the three adjacencies corresponding to the dotted orange edges.

This problem can be solved independently and recursively for each AB-cycle. For an added adjacency *a*, the AB-cycle is divided in two and the algorithm is then executed on both cycles. This is the Guided IG function from Algorithm 1, with a small example shown in Figure 8.

An interesting property of this algorithm is that it can introduce new adjacencies, other than the ones from the guide *G*, when this is the only option for an intermediate genome. Specifically, for any AB-cycle of size 2, either from the original BP(*A, B*) or coming from the recursion, the corresponding adjacency is chosen, since there is no other option, as *M *has to be an intermediate genome. For instance, in Figure 8, although only two adjacencies from the guide are used, it is possible to infer three more, because of the three cycles of size 2.

The next question is then, what to do with the larger AB-cycles? One can think of two ways to answer this question, based on the different approach between *homology-based *and *distance-based *algorithms.

As an homology-based algorithm, after the guide is processed, no new adjacency is added in *M *, since the aim is to reconstruct contiguous ancestral regions (CARs), without the need for a complete genome, and there is not enough information to choose new adjacencies. If we think as a distance algorithm, trying to minimize the tree length, it is not a good idea to have a fragmented genome, and completing the adjacencies in any non-crossing way builds an intermediate genome *M *, minimizing *d*(*A*, *M*) + *d*(*B*, *M*). Since at this point in the algorithm we have no extra information, one way would be to just choose non-crossing edges randomly. In practice the adjacencies were chosen all from the same colored edges, either all A-edges or all B-edges, in an attempt to maximize the common adjacencies with input genomes *A *and *B*. The number of common adjacencies does not correspond directly to the number of A- or B-edges in a component, since the recursion produces new A- and B-edges that might not be present in the original genomes. From the two possible ways to do that (all A- or all B-edges), we choose the one with more common adjacencies. For instance, on the AB-cycle of Figure 8, we can choose either the two green or two blue edges, but both blue ones are original from *B*, and only one green is from *A*, therefore we choose the blue adjacencies. If there is no difference, one option is picked randomly.

A pseudo-code of the main functions of the ancestral reconstruction algorithm is shown in Algorithm 1.

#### Finding adjacency guides

How do we find adjacency guides? The simplest answer is to search directly in the tree, traversing it from the internal node being reconstructed. For each leaf found, from closest to farthest, its adjacencies are added to the guide. It is a very naive algorithm but, somewhat surprisingly, it can give good results. This guide will be called *leaf guide*.

Better adjacency guides potentially improve the reconstruction, and there is already a good amount of well designed algorithms for finding those guides. Namely, the output of any homology algorithm, such as InferCARs [9], ANGES [11], SCJ [7] or Pro-CARs [14], can be directly used as a guide. It does not mean that all adjacencies from the homology algorithm are automatically added to the ancestral genome; each adjacency is still going to be considered and possibly rejected if it breaks any intermediate genome property. This type of guide is the *ancestral guide*.

Any combination of ancestral guides and leaf guide could potentially be used. In the Results section a more detailed description of the combinations tested in this work is given.

## Results

The proposed algorithms were implemented in Python and tested on simulated datasets, where a tree with genomes at the leaves is given as input, and the algorithms try to reconstruct the ancestral genomes at internal nodes of the tree. The amount of correct adjacencies, missing adjacencies and wrong adjacencies for each ancestral genome is then measured.

To compare the proposed algorithms, as the homology methods representatives we ran SCJ [7] and Pro-CARs [14]. SCJ was chosen because since it is a very conservative method, it has a very low false positive rate [7], providing an almost error-free set of adjacencies that the intermediate genomes method can improve upon. ProCARs was chosen because it was the easiest of the homology methods to run, and according to its authors it has similar results when compared to ANGES, InferCARs and GapAdj [14]. For the distance methods, PATHGROUPS [5] and GASTS [6] were chosen, as these are the most recent methods for DCJ-based small phylogeny.

For the Intermediate Genomes algorithm, four variations of ancestral guides were tested: no ancestral guide, SCJ, ProCARs and *perfect*. The perfect guide is a "cheating algorithm" that uses as guide the correct ancestral genome, from the simulation. This is done to determine some form of "upper bound" on the proposed IG algorithms, as we can measure how many adjacencies are rejected by the algorithm, even though they are present in the correct ancestral genome. On all four types, after all adjacencies from the ancestral guide are applied, the leaf guide is used. These four variants will be called IG-Pure, IG-SCJ, IG-ProCARS and IG-perfect, depending on the ancestral guide.

For each of these four guide options, two variants were tested: the *homology *approach, were no extra adjacencies are added in the guided IG problem, for components larger than 2; and the *distance *approach, were adjacencies are added, and since there is some randomization in the choice, the algorithm runs 50 times, with the shortest DCJ distance tree chosen as the output.

1: **function **IG_SMALL_PHYLOGENY(T,G)

2:   **while **|*T*| > 2 **do**

3:      *n*_1_, *n*_2 _← find closest_leaves(*T*)

4:      *p *← parent(*n*_1_, *n*_2_)

5:      *G *← GET_NODE_GUIDE (*p*)

6:      $G\left[p\right]\leftarrow$ ← GUIDED_IG(*G*,  G[*n*_1_],  G[*n*_2_])

7:      *T*.prune(*n*_1_, *n*_2_)                     ⊲ *p *becomes a leaf.

8:   **return ** G

9: **function **GUIDED_IG(*G, A, B*)

10:   *I *← ∅

11:   **for ***C *∈ BP(*A, B*) **do**

12:      *I *← *I *∪ GUIDED_IG_C(*G*, *C*)

13:   **return **GENOME(*I*)

14: **function **GUIDED_IG_C(*G, C*)

15:   **if ***C *= ∅ **then**: **return **∅

16:   *n *← |*C*|

17:   **if ***n *= 2 **then**: **return ***C*

18:   **while ***G *≠ ∅ **do**

19:      *pq *← pop_first_element(*G*)

20:      **if ***p *∉ or *q *∉ *C ***then**: **continue**

21:      *i *← *C*.idx(*p*)

22:      *j *← *C*.idx(*q*)

23:      **if **(*j *− *i*) mod 2 = 0 **then**: **continue**

24:      *A*_1 _= GUIDED_IG_C(*G, C*[*i *+ 1,..., *j *− 1])

25:      *A*_2 _= GUIDED_IG_C(*G, C*[1,..., *i*−1]∪*C*[*i*+1,..., *n*])

26:      **return ***{pq} *∪ *A*_1 _∪ *A*_2_

27:   **if **HOMOLOGY **then**: **return **∅

28:   *S*_1 _← {{*C*[1], *C*[2]}, {*C*[3], *C*[4]},... {*C*[*n *− 1], *C*[*n*]}}

29:   *S*_2 _← {{*C*[2], *C*[3]}, {*C*[4], *C*[5]},... {*C*[*n*], *C*[1]}}

30:   **return **$\underset{S\in \{S_{1},S_{2}\}}{\arg\max}(|S\cap A|+|S\cap B|)$

**Algorithm 1: **Ancestral Reconstruction with Intermediate Genomes. The main function is IG_SMALL_PHYLOGENY, that receives a tree *T *and a genome list  G, at the leaves of *T *, and returns a new list with added ancestral genomes. In a bottom-up approach, it chooses two leaves, to reconstruct the ancestral parent node. First, it builds an adjacency guide with ancestral and/or leaf guides, as described in the text. Then it calls GUIDED_IG, that will build a set of adjacencies of the ancestral genome by calling GUIDED_IG_C for each component of BP(*A, B*), which in turn calls itself recursively each time an adjacency is applied in a component. When there are no adjacency guides for a component, in the homology approach no new adjacency is returned; in the distance approach, two sets are built, with all adjacencies from the same color, and the one with more common adjacencies with the input genomes is returned. After an ancestral genome is reconstructed, its leaves are pruned so it becomes a leaf and the main loop continues until all the ancestral genomes have been reconstructed.

### Simulated datasets

For each simulation, similarly as previous works [24], a birth-death model with a birth rate of 0.001 and a death rate of 0 generates an ultrametric tree with a chosen number of leaves. To modify the branch lengths, for each branch a real number *d *is uniformly chosen from the interval [−*c*, +*c*], and the branch length is multiplied by *e^d ^*. For this experiments, *c *= 2. The branch lengths are then rescaled so the tree has a target diameter *D*. At the root of the tree, a genome is generated and evolved through the tree, applying *l *rearrangements in each branch, where *l *is the branch length. The rearrangement events are randomly chosen between reversals (90%), transpositions (5%) and translocations (5%). We generated 2 main datasets, one with 20 leaf genomes with 500 genes, and another with 12 leaf genomes with 5000 genes. Also, the diameter *D *varies from 1*n *to 4*n*, where *n *is the number of genes. For each *D*, 10 simulations were generated. The leaf genomes and the tree topology is then given as input for the ancestral reconstruction methods.

## Discussion

**Figure 9:**
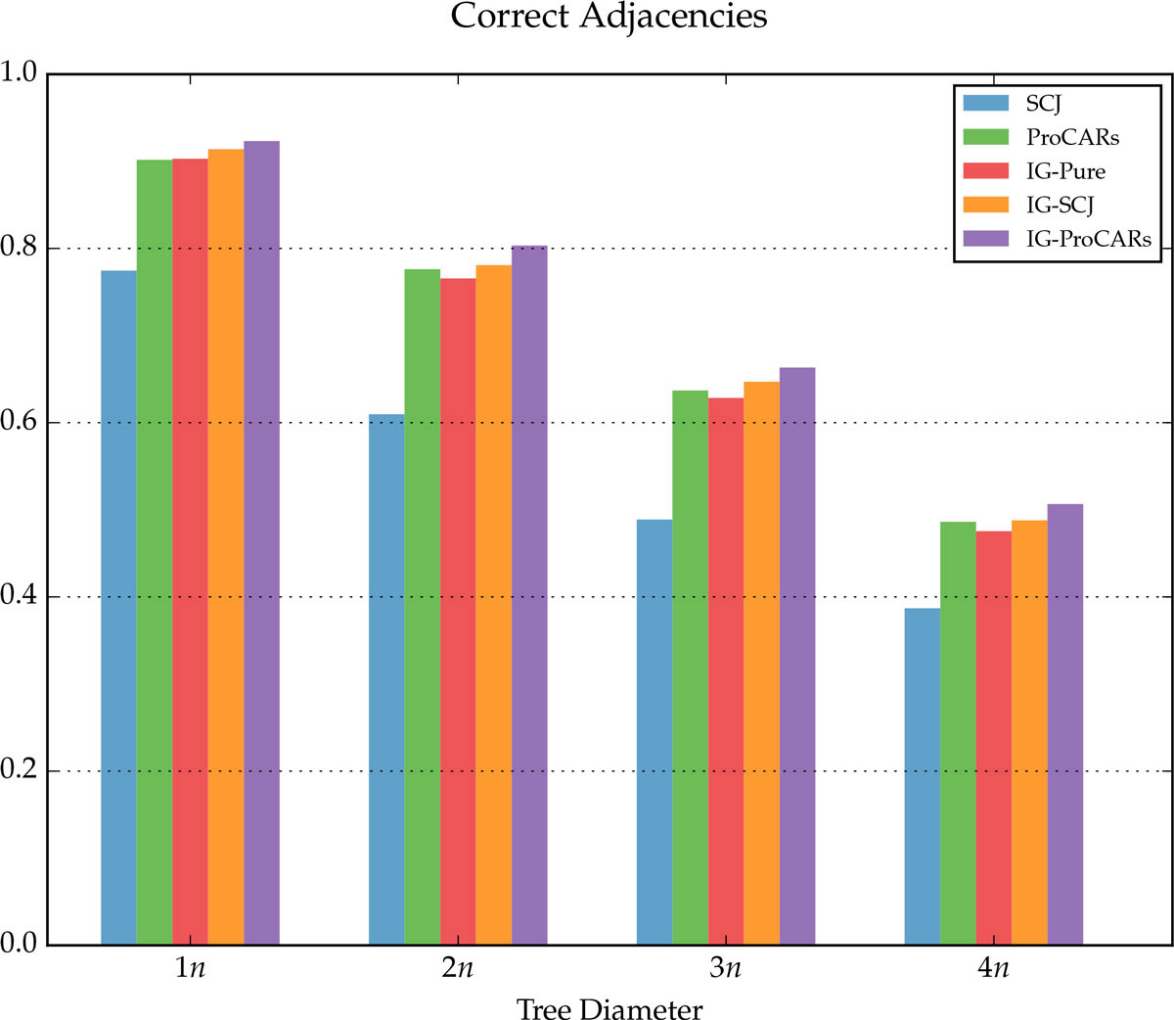
**Percentage of correct adjacencies found by the Homology algorithms, for each tree diameter**.

**Figure 10:**
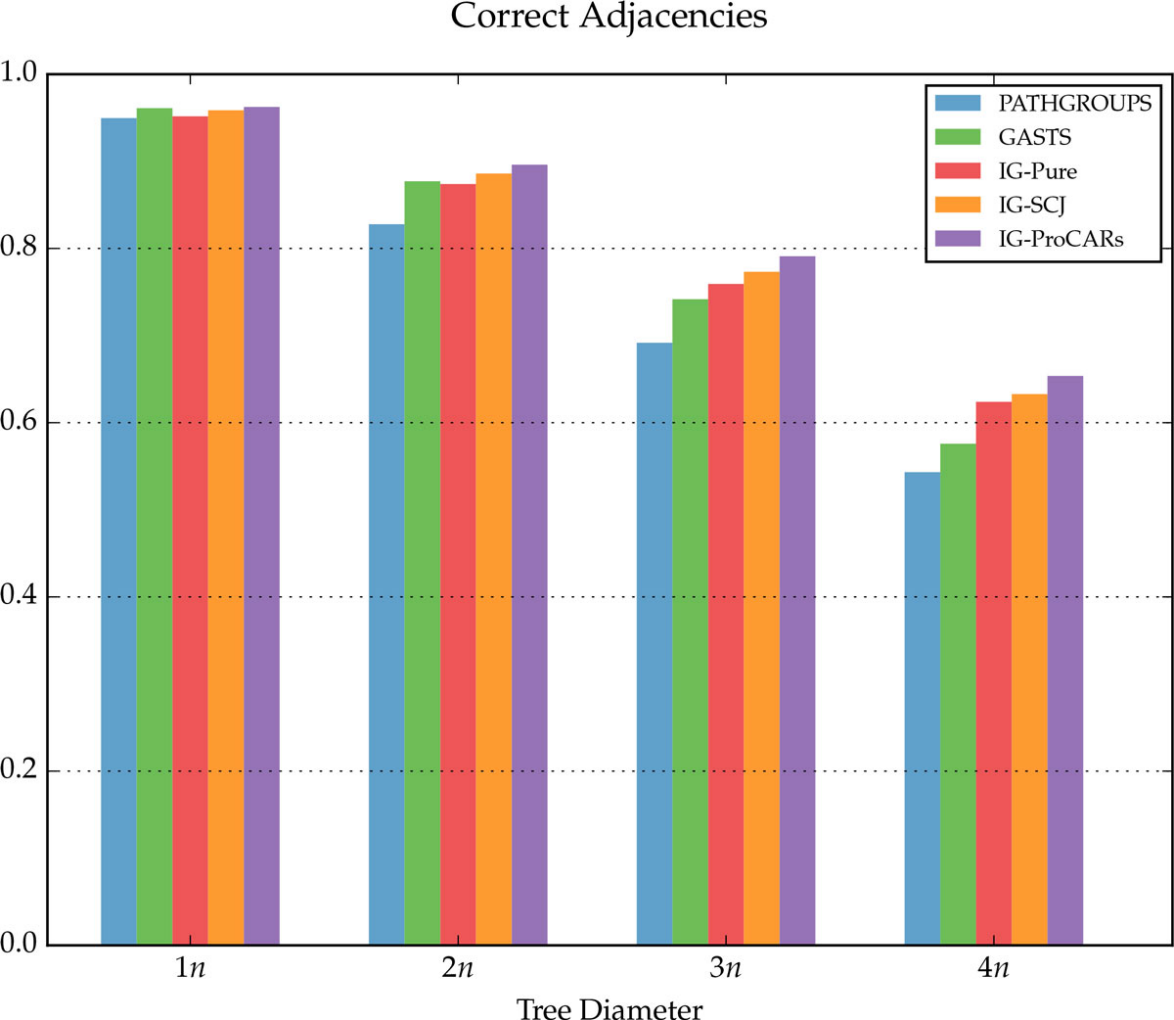
**Percentage of correct adjacencies found by the Distance algorithms, for each tree diameter**.

**Figure 11:**
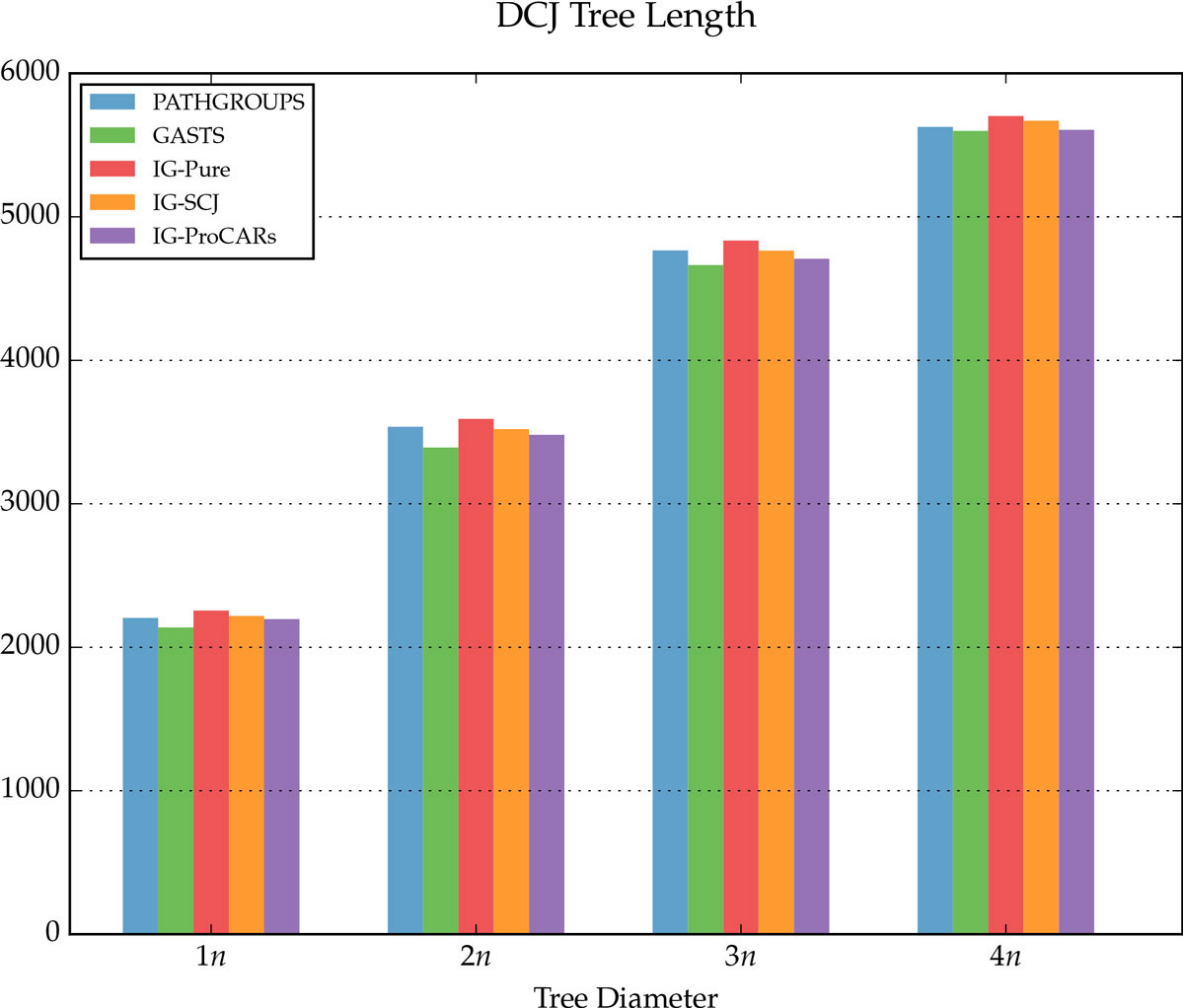
**Total DCJ tree length for the distance algorithms**.

**Figure 12:**
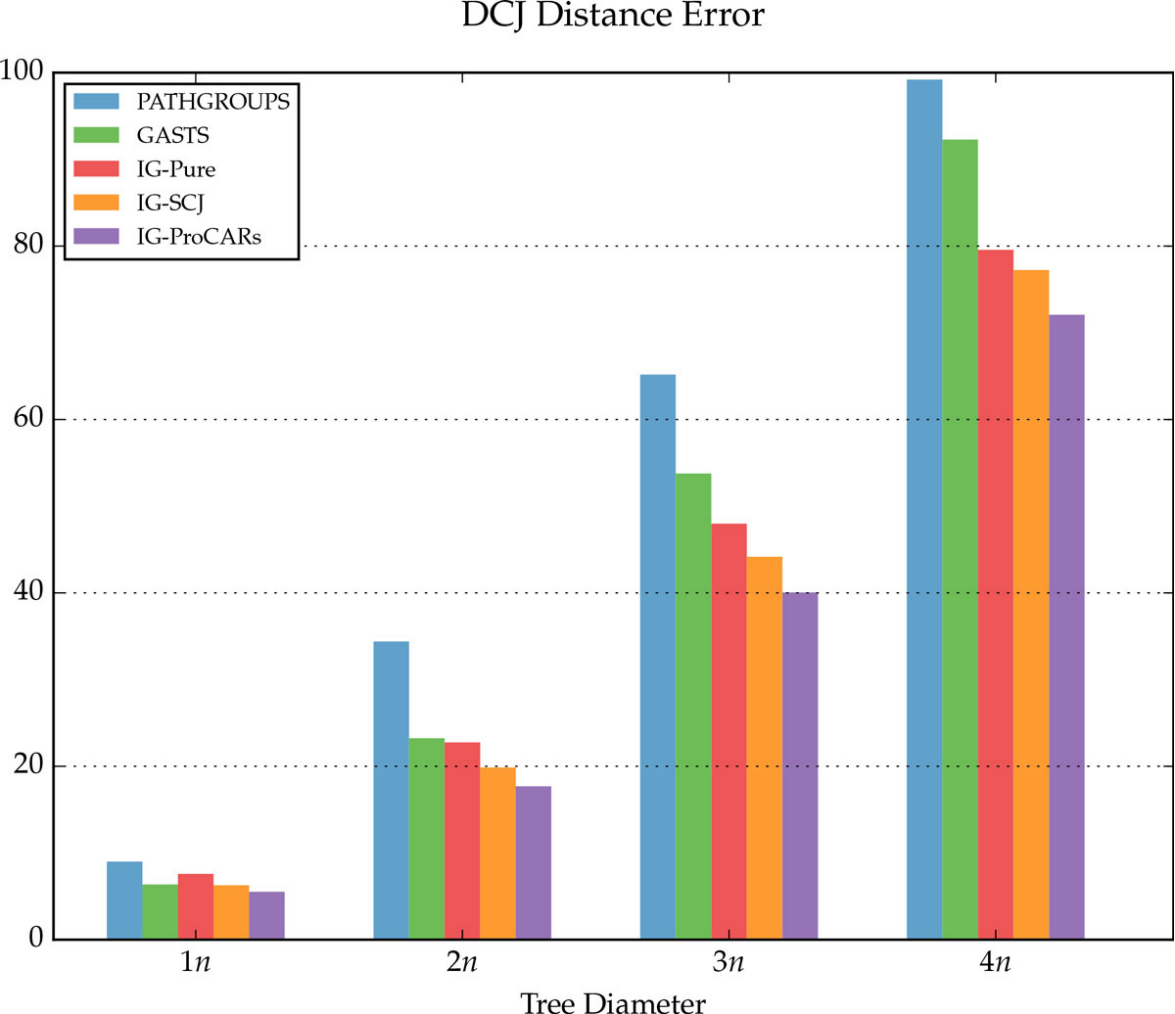
**Total DCJ distance between the simulated and reconstructed ancestors for the distance algorithms**.

The simulation results are summarized in Figure 9, for the homology algorithms, and in Figures 10, 11 and 12, for the distance algorithms. More detailed results are shown in Table 1 and running times for two different datasets are displayed in Table 2.

**Table 1:** Summary of the results for each algorithm, in the 20 genomes with 500 genes dataset.

Diameter	*D *= 1*n*			*D *= 2*n*			*D *= 3*n*			*D *= 4*n*		
				
Results (%)	TP	FN	FP	TP	FN	FP	TP	FN	FP	TP	FN	FP
*Homology methods*												
SCJ	77.5	21.5	0.0	61.0	38.0	0.0	48.9	50.1	0.0	38.7	60.3	0.1
ProCARs	90.2	8.8	0.4	77.6	21.4	0.7	63.7	35.3	1.2	48.6	50.4	1.9
IG-Pure	90.3	8.7	0.2	76.6	22.4	0.5	62.8	36.2	1.0	47.5	51.5	1.9
IG-SCJ	91.4	7.6	0.2	78.1	20.9	0.5	64.7	34.3	1.1	48.8	50.2	1.9
IG-ProCARs	92.3	6.7	0.3	80.3	18.7	0.7	66.3	32.7	1.6	50.7	48.3	2.8
IG-Perfect	95.4	3.6	0.1	90.5	8.5	0.2	82.0	17.0	0.3	71.7	27.3	0.5
*Distance methods*												
GASTS	95.6	3.4	3.4	87.3	11.7	11.7	73.8	25.2	25.2	57.3	41.7	41.7
PATHGROUPS	94.7	4.3	4.7	82.6	16.4	16.9	69.0	30.0	30.5	54.2	44.8	45.3
IG-Pure	94.7	4.3	4.3	87.1	11.9	12.1	75.7	23.3	23.6	62.2	36.8	37.2
IG-SCJ	95.4	3.6	3.7	88.3	10.7	10.9	77.0	22.0	22.2	63.1	35.9	36.3
IG-ProCARs	95.8	3.2	3.3	89.3	9.7	9.9	78.8	20.2	20.4	65.1	33.9	34.2
IG-Perfect	96.7	2.3	2.4	93.3	5.7	5.8	86.0	13.0	13.3	75.4	23.6	24.0

Each number is the average of 10 simulations, for 4 different tree diameters. The columns show the percentage of *true positives *(correctly reconstructed adjacencies), *false negatives *(missing adjacencies, present in true ancestral but not on the reconstructed genome) and *false positives *(wrong adjacencies, present in the reconstructed genome but not on the true ancestral).

**Table 2:** Average running time of 10 runs of each algorithm, for different tree diameters in two different simulated datasets.

Dataset	20 genomes, 500 genes				12 genomes, 5000 genes			
		
Diameter	*D *= 1*n*	*D *= 2*n*	*D *= 3*n*	*D *= 4*n*	*D *= 1*n*	*D *= 2*n*	*D *= 3*n*	*D *= 4*n*
SCJ	3 s	3 s	4 s	4 s	7 s	9s	11 s	11 s
PATHGROUPS	18 s	40 s	57 s	1 m18 s	22 m16 s	1 h21 m42 s	1 h16 m34 s	31 m13 s
GASTS	22 s	1 m6 s	2 m46 s	4 m41 s	1 h14 m6 s	12 h27 m13 s	19 h8 m55 s	22 h2 m32 s
ProCARs	14 m9 s	30 m10 s	36 m28 s	1 h9 m5 s	-	-	-	-
IG-Pure	2 s	3 s	3 s	3 s	1 m38 s	2 m23 s	2 m44 s	1 m53 s
IG-SCJ	2 s	3 s	4 s	4 s	1 m4 s	1 m41 s	2 m8 s	1 m26 s
IG-ProCARs	3 s	3 s	4 s	4 s	-	-	-	-

In the combined IG algorithms, only the IG time is shown, without the homology method time (SCJ or ProCARs). Also, for the IG-methods ran as distance-based algorithms, the time is roughly 50× more, since the algorithms were repeated 50 times and the smallest tree was chosen.

For each internal node of the tree, given the reconstructed genome *R *and the simulated ancestral *S*, three measurements were made: *correct adjacencies *(true positives in Table 1), adjacencies in *R *also present in *S*; *missing adjacencies *(false negatives in Table 1), present in *S *but not in *R*; and *wrong adjacencies *(false positives in Table 1), present in *R *but not in *S*. The percentage of all adjacencies in each category, for each different tree diameter *D*, is shown in the figures and table. For the distance methods, the total DCJ length of the tree is also measured, as well as the DCJ error, the distance between *R *and *S*, and the total sum of these values is shown on Figure 10.

The homology algorithms have a very low error rate, because they tend to choose adjacencies only when there is good information for doing it. In contrast, distance algorithms usually have a higher number correct adjacencies, at the cost of a much larger error rate, since they have to build more complete genomes that minimize distances, filling the missing adjacencies.

For the homology algorithms comparison, we can see that the IG-Pure algorithm already has good results in terms of correctly reconstructed adjacencies, almost the same as ProCARs. When combined with SCJ and ProCARs as ancestral guide, the IG algorithm improves on both original algorithms, with only a slight increase in wrong adjacencies.

Compared to the distance methods, the IG algorithms have better results in almost all measurements, especially as the tree diameter increases. While the DCJ tree length from PATHGROUPS and GASTS is always smaller on average, which is expected since they are distance minimizers, the IG algorithms are quite close, even though they are not designed to minimize the tree length, and in a few instances the IG-ProCARs algorithm even returned a smaller tree. More interestingly, the DCJ error between the reconstructed and real ancestral genomes is smaller in the IG methods, significantly more as the diameter increases, raising the question: is it the best choice to find a genome median, minimizing the length of the tree, when this median is actually more distant to the true ancestral genome?

On the other hand, if we focus on the IG-perfect results shown in Table 1 we can identify the proportion of adjacencies from the true ancestral genomes that were rejected because they are not part of the intermediate genome being reconstructed. The more distant the genomes are, the more adjacencies are rejected, with almost 30% of the adjacencies of the true ancestors being rejected when the diameter is 4*n*. This shows that even though restricting intermediate genomes on internal nodes seems to enhance results over the unrestricted versions, there is still much to be improved. An open question is whether a mixed approach can be developed, allowing some flexibility on departing from the intermediate genome restriction, potentially obtaining better results and, perhaps the more challenging aspect, how can we detect when to do it?

## Conclusion

A new approach for ancestral reconstruction of gene orders is proposed. The key aspect is the restriction that each ancestral genome must be an intermediate genome of its children. This would seem like a basic assumption in parsimonious methods, but it was not used in previous methods. The results of this paper indicate that, even with naive approaches, simple algorithms based on intermediate genomes are fast and obtain good results. Furthermore, they are easily combined with homology-based algorithms, enhancing their results.

There are many directions for improving the current approach, as many simple algorithm design decisions were made in this first study. For instance, the order of the adjacencies in the guides is definitely important in the reconstructed ancestral genomes, as it potentially changes which adjacencies are accepted or rejected. A better logic in selecting which adjacencies are used and in which order they should be considered might increase the number of correctly reconstructed adjacencies. For the distance-based approach, when adjacencies have to be included without guide information, instead of the simple approach presented here, a better option would be to choose intermediate genomes that try to respect the distance given by the tree branch lengths, if available.

In a more high level view, it would be interesting to investigate the concept of intermediate genomes in more complex DCJ models, that include insertion and deletions events [25,26], to develop more complete ancestral reconstruction methods, that can accept genomes with different sets of genes, a limitation of any parsimonious method based on the basic DCJ distance.

## Competing interests

The authors declare that they have no competing interests.

## References

[B1] Sankoff D Blanchette M Multiple genome rearrangement and breakpoint phylogeny Journal of Computational Biology 1998 5 3 555 570 10.1089/cmb.1998.5.555 9773350

[B2] Yancopoulos S Attie O Friedberg R Efficient sorting of genomic permutations by translocation, inversion and block interchange Bioinformatics 2005 21 16 3340 6 10.1093/bioinformatics/bti535 15951307

[B3] Bergeron A Mixtacki J Stoye J A unifying view of genome rearrangements Lecture Notes in Computer Science 2006 4175 163 173 10.1007/11851561_16

[B4] Alekseyev MA Pevzner PA Breakpoint graphs and ancestral genome reconstructions Genome Research 2009 19 5 943 57 10.1101/gr.082784.108 19218533 PMC2675983

[B5] Zheng C Sankoff D On the PATHGROUPS approach to rapid small phylogeny BMC bioinformatics 2011 12 Suppl 1 4 10.1186/1471-2105-12-S1-S4 21342571 PMC3044296

[B6] Xu W Moret B GASTS: Parsimony scoring under rearrangements Proceedings of the 11th International Workshop on Algorithms in Bioinformatics (WABI 2011) 2011 351 363

[B7] Biller P Feijão P Meidanis Ja Rearrangement-based phylogeny using the single-cut-or-join operation IEEE/ACM Transactions on Computational Biology and Bioinformatics 2013 10 1 122 134 23702549 10.1109/TCBB.2012.168

[B8] Feijao P Meidanis J SCJ: a breakpoint-like distance that simplifies several rearrangement problems IEEE/ACM Transactions on Computational Biology and Bioinformatics 2011 8 1318 1329 21339538 10.1109/TCBB.2011.34

[B9] Ma J Zhang L Suh BBB Raney BJBJ Burhans RC Kent WJJ Blanchette M Haussler D Miller W Reconstructing contiguous regions of an ancestral genome Genome Research 2006 16 12 1557 1565 10.1101/gr.5383506 16983148 PMC1665639

[B10] Gagnon Y Blanchette M El-Mabrouk N A flexible ancestral genome reconstruction method based on gapped adjacencies BMC bioinformatics 2012 13 Suppl 1(Suppl 19) 4 23281872 10.1186/1471-2105-13-S19-S4 PMC3526437

[B11] Jones BR Rajaraman A Tannier E Chauve C ANGES: reconstructing ANcestral GEnomeS maps Bioinformatics (Oxford, England) 2012 28 18 2388 2390 10.1093/bioinformatics/bts457 22820205

[B12] Yang N Hu F Zhou L Tang J Reconstruction of ancestral gene orders using probabilistic and gene encoding approaches PloS one 2014 9 10 108796 10.1371/journal.pone.0108796 PMC4193752 25302942

[B13] Hu F Lin Y Tang J MLGO: phylogeny reconstruction and ancestral inference from gene-order data BMC bioinformatics 2014 15 1 354 25376663 10.1186/s12859-014-0354-6 PMC4236499

[B14] Perrin A Varré J-s Blanquart S Ouangraoua A ProCARs : Progressive Reconstruction of Ancestral Gene Orders BMC Genomics 2015 16 Suppl 5 6 10.1186/1471-2164-16-S5-S6 26040958 PMC4460626

[B15] Braga MDV Stoye J The solution space of sorting by DCJ Journal of Computational Biology 2010 17 9 1145 65 10.1089/cmb.2010.0109 20874401

[B16] Tannier E Zheng C Sankoff D Multichromosomal median and halving problems under different genomic distances BMC bioinformatics 2009 10 120 10.1186/1471-2105-10-120 19386099 PMC2683817

[B17] Ouangraoua A Bergeron A Combinatorial structure of genome rearrangements scenarios Journal of computational biology : a journal of computational molecular cell biology 2010 17 9 1129 1144 doi:10.1089/cmb.2010.0126 10.1089/cmb.2010.0126 20874400

[B18] The On-Line Encyclopedia of Integer Sequences 2010 Accessed: 2015-06-14

[B19] Xu AW The median problems on linear multichromosomal genomes: graph representation and fast exact solutions Journal of Computational Biology 2010 17 9 1195 211 10.1089/cmb.2010.0106 20874404

[B20] Zhang M Arndt W Tang J An exact solver for the DCJ median problem Pacific Symposium on Biocomputing 2009 138 49 19209699 PMC2792274

[B21] Haghighi M Sankoff D Medians seek the corners, and other conjectures BMC bioinformatics 2012 13 Suppl 1(Suppl 19) 5 23281922 10.1186/1471-2105-13-S19-S5 PMC3526443

[B22] Swenson KM Moret BME Inversion-based genomic signatures BMC bioinformatics 2009 10 Suppl 1 7 doi:10.1186/1471-2105-10-S1-S7 10.1186/1471-2105-10-S1-S7 19208174 PMC2648787

[B23] Swenson KM To Y Tang J Moret BME Maximum independent sets of commuting and noninterfering inversions BMC bioinformatics 2009 10 Suppl 1 6 doi:10.1186/1471-2105-10-S1-S6 10.1186/1471-2105-10-S1-S6 19208163 PMC2648783

[B24] Lin Y Hu F Tang J Moret B Maximum Likelihood Phylogenetic Reconstruction from High-Resolution Whole-Genome Data and a Tree of 68 Eukaryotes Pacific Symposium on Biocomputing 2013 285 296 23424133 PMC3712796

[B25] Braga MDV Willing E Stoye J Double cut and join with insertions and deletions Journal of Computational Biology 2011 18 9 1167 84 doi:10.1089/cmb.2011.0118 10.1089/cmb.2011.0118 21899423

[B26] Compeau PE DCJ-Indel sorting revisited Algorithms for molecular biology : AMB 2013 8 1 6 doi:10.1186/1748-7188-8-6 10.1186/1748-7188-8-6 23452758 PMC3655023

